# Risk factors for incident anemia of chronic diseases: A cohort study

**DOI:** 10.1371/journal.pone.0216062

**Published:** 2019-05-06

**Authors:** Yun-Gyoo Lee, Yoosoo Chang, Jihoon Kang, Dong-Hoe Koo, Seung-Sei Lee, Seungho Ryu, Sukjoong Oh

**Affiliations:** 1 Department of Internal Medicine, Kangbuk Samsung Hospital, Sungkyunkwan University School of Medicine, Seoul, Korea; 2 Department of Occupational and Environmental Medicine, Kangbuk Samsung Hospital, Sungkyunkwan University, School of Medicine, Seoul, Korea; 3 Center for Cohort Studies, Total Healthcare Center, Kangbuk Samsung Hospital, Sungkyunkwan University, School of Medicine, Seoul, Korea; Universita degli Studi di Perugia, ITALY

## Abstract

**Objective:**

Anemia of chronic disease (ACD) refers to hypoproliferative anemia in the context of acute or chronic activation of the immune system. There is a paucity of prospective data addressing the risk factors for ACD development. An association between common chronic diseases and ACD was examined cross-sectionally and longitudinally.

**Method:**

A cohort of 265,459 healthy participants without ACD at baseline were prospectively followed annually or biennially.

**Results:**

During average follow-up period of 62 months, 4,906 participants developed ACD (incidence rate 3.58 per 1000 person-years). Multivariable-adjusted hazard ratio (HR) [95% confidence interval (CI)] for incident ACD comparing estimated glomerular filtration rate 30–60 and < 30 vs. ≥ 60 ml/min/1.73 m^2^ were 3.93 [3.18–4.85] and 39.11 [18.50–82.69]; HRs [95% CI] for ACD comparing prediabetes and diabetes vs. normal were 1.19 [1.12–1.27] and 2.46 [2.14–2.84], respectively. HRs [95% CI] for incident ACD comparing body-mass-index (BMI) of < 18.5, 23–24.9 and ≥ 25 vs. 18.5–22.9 kg/m^2^ were 0.89 [0.78–1.00], 0.89 [0.80–0.99] and 0.78 [0.66–0.91], respectively. HRs [95% CI] for incident ACD comparing prehypertension and hypertension vs. normal were 0.79 [0.73–0.86] and 1.10 [0.99–1.23], respectively. Metabolic syndrome, hypertension, chronic liver disease, and chronic obstructive pulmonary disease were not associated with incident ACD.

**Conclusions:**

The severity of chronic kidney disease and diabetic status were independently associated with an increased incidence of ACD, whereas prehypertension and an increasing BMI were significantly associated with decreased risk of ACD.

## Introduction

Anemia of chronic disease (ACD) refers to normochromic, normocytic, hypoproliferative anemia in the context of acute or chronic inflammatory states, including infections, cancers, and autoimmune conditions.[1, 2] Some epidemiological studies have reported that ACD also occurs in clinical conditions accompanied by mild but persistent inflammation including chronic kidney disease (CKD), diabetes mellitus, and aging.[3–5] The prevalence of anemia from most causes has decreased globally between 1990 and 2010, but ACD is expected to increase as population ages.[6–8]

Although the underlying pathophysiology of ACD is multifactorial, hepcidin may play a central role in ACD.[9] Chronic inflammation elevates pro-inflammatory cytokines, including interleukin-6, which centrally mediates hepcidin synthesis. Hepcidin inhibits iron absorption in the intestine and release of recycled iron from macrophages, resulting in reduced efficiency of iron recycling from red blood cells. This functional iron deficiency leads to impaired proliferation of erythroid progenitor cells in the marrow, resulting in iron-restrictive anemia.[3]

ACD is common but often overlooked in actual clinical practice and the risk factors of ACD is not fully understood. CKD leads to dysfunction of renal erythropoietin-producing cells resulting in normocytic normochromic anemia, which was present in nearly half of patients with CKD.[10, 11] Type 2 diabetes increases the risk for anemia by two or three times, which affects 10–15% of patients with type 2 diabetes.[12–14] In these studies, anemia in diabetic patients can be considered as ACD, including the exclusion of iron deficiency anemia and other causes of secondary influences on hemoglobin levels.[14] ACD is also frequently diagnosed in the elderly (>65 years); a few population-based studies have shown that 17% of the elderly are anemic,[15] and 70% of hospitalized elderly patients with anemia were found to have ACD.[5] However, most studies focused on specific single disease or elderly population and were cross-sectional studies limited by the temporal ambiguity between risk factors and anemia. Until now, there is a paucity of prospective cohort study to demonstrate the risk factors for the development of ACD in general population. We examined a prospective relationship of common chronic diseases and their severity with the development of ACD in a large cohort of young and middle-aged Korean adults who underwent a regular health screening examination.

## Patients and methods

### Study population

**Fig 1:**
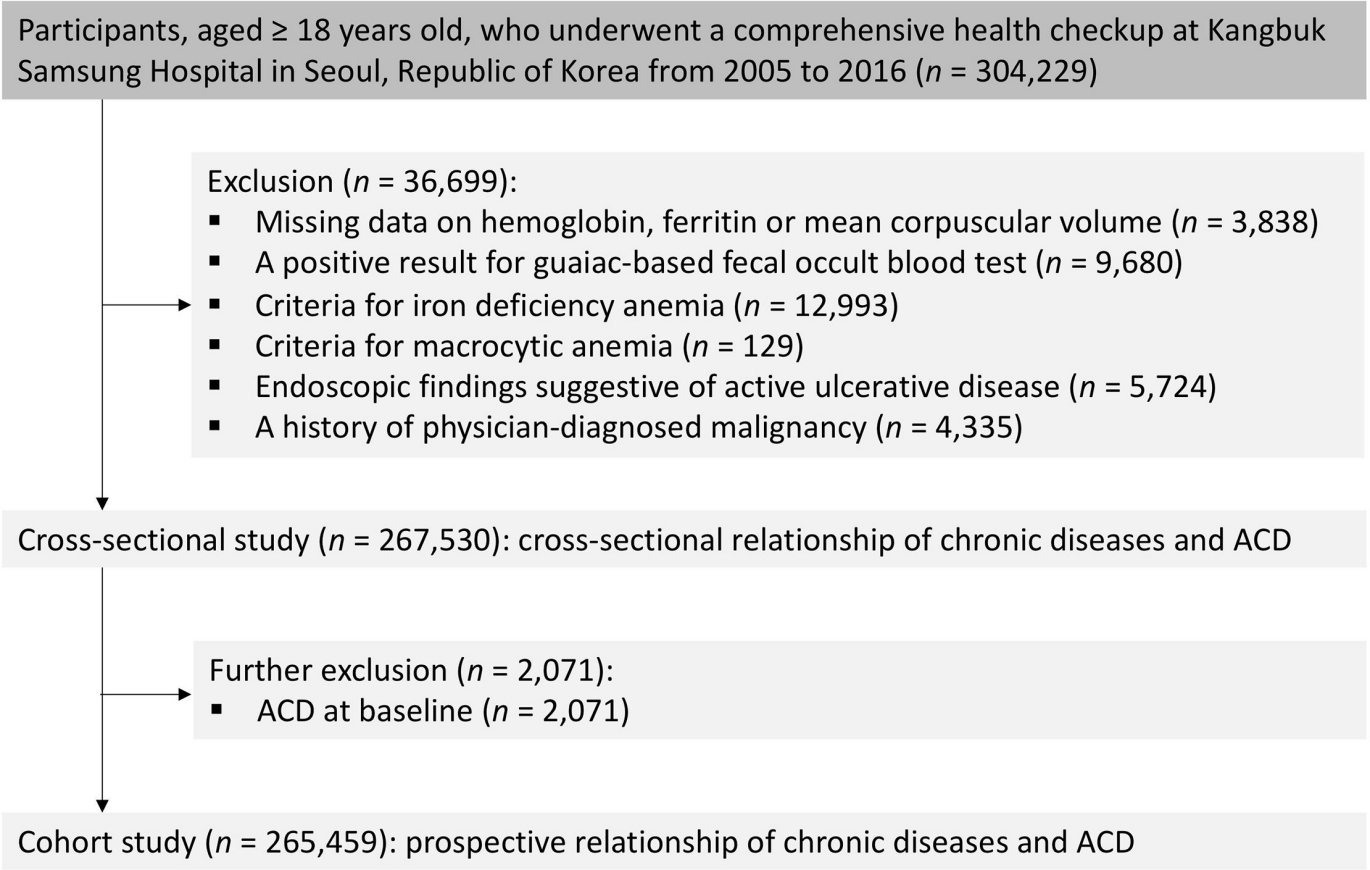
Flow chart of study population.

The Kangbuk Samsung Health Study (KSHS) is a cohort study of Korean men and women men and women ≥ 18 years of age who underwent a comprehensive regular (annual or biennial) health examination at Kangbuk Samsung Hospital Total Healthcare Centers in Republic of Korea.[16] The current analyses included all study participants with at least one follow-up visit who underwent a comprehensive health examination between 2005 and 2015 and were followed annually or biennially until December 2016 (n = 304,229). ACD was defined as having anemia without evidence of nutritional anemia or gastrointestinal blood loss.[9] The selection process of study participants is shown in Fig 1. We excluded subjects with missing data on hemoglobin, ferritin, or mean corpuscular volume (MCV) at baseline (n = 3,838), subjects with positive for fecal occult blood tests (n = 9,680), subjects with iron deficiency anemia (n = 12,993) or macrocytic anemia (n = 129), subjects with endoscopic findings including gastric ulcers, duodenal ulcer, inflammatory bowel disease, angiodysplasia, or other gastrointestinal malignancies (esophageal cancer, gastric cancer, or colorectal cancer) (n = 5,724), or subjects with a history of physician-diagnosed malignancy (n = 4,335). After excluding 36,699 subjects, 267,530 participants were included in the baseline cross-sectional study. The cohort participants were not registered at once, but were made up of KSHS cohort in the form of new subjects being added each year. (S1 Table) For cohort study, we further excluded an additional 2,071 subjects with ACD at baseline and finally analyzed 265,459 subjects.

### Measurements

Anthropometric measurements and procedures for obtaining blood samples were described previously.[16, 17] In accordance with WHO criteria, anemia was defined as a hemoglobin level < 13.0 g/dL in men and < 12.0 g/dL in women.[18, 19] Iron deficiency was defined as anemia with a transferrin saturation rate < 16%, or a serum ferritin concentration < 30 ng/mL. Transferrin saturation was calculated by dividing serum iron by total iron-binding capacity. Macrocytic anemia was defined as anemia with MCV > 100 fL.[20] CKD was defined as estimated glomerular filtration rate (eGFR) was <60 ml/min/1.73 m^2^. Body mass index (BMI) was categorized according to Asian-specific criteria;[21] underweight, BMI < 18.5 kg/m^2^; normal weight, BMI of 18.5–22.9 kg/m^2^; overweight, BMI of 23–24.9 kg/m^2^; and obese, BMI ≥ 25 kg/m^2^. Metabolic syndrome (MetS) was defined using harmonized criteria.[22, 23] Blood pressure (BP) was categorized into normal BP (BP of <120/<80mmHg), prehypertension (systolic BP of 120–139 mmHg or diastolic BP of 80–89 mmHg) and hypertension (systolic BP of ≥140mmHg or diastolic BP of ≥90 mmHg). Diabetes mellitus was defined as either fasting serum glucose ≥126 mg/dL, glycated hemoglobin (HbA1c) ≥ 6.5%, or the use of blood glucose-lowering drugs, and prediabetes was defined as either fasting serum glucose 100–125 mg/dL or HbA1c 5.7–6.4%. Subjects were considered to have chronic liver disease if either serum hepatitis B surface antigen or anti-hepatitis C antibody was positive, or they had an ultrasonographic diagnosis of fatty liver, or liver cirrhosis. Chronic obstructive pulmonary disease (COPD) was defined as forced expiratory volume in 1 sec/forced vital capacity ratio <0.7.

### Statistical analyses

We first evaluated the cross-sectional relationship between comorbidities and ACD at baseline, and then we analyzed the prospective relationship of comorbidities and incident ACD in a cohort study. To compare the characteristics of the study participants between the groups, we used an independent t-tests for continuous variables or χ^2^ tests for categorical variables. To determine the cross-sectional relationship between comorbidities and ACD, we used a logistic regression model to estimate odds ratios (ORs) with 95% confidence interval (CI). Then, the hazard ratio (HR) and 95% CI were calculated for incident ACD according to comorbidities. Each participant was followed from baseline exam until either development of ACD or the last health exam conducted prior to December 31, 2016, whichever came first. The incidence rate was calculated as number of incident cases divided by number of person-years of follow-up. Since ACD was known to have developed between the two visits, but the precise time at which it developed was unknown, a parametric proportional hazard model was used to account for this type of interval censoring (*stpm* command in Stata). To determine linear trends of incidence, the number of categories was used as a continuous variable and tested on each model.

The models were initially adjusted for age and sex, and then were adjusted for smoking, alcohol intake, physical activity, education level, center, and year of screening examination. All analyses were performed using STATA version 15.0 (StataCorp, College Station, Texas, USA).

### Ethics

This study was approved by the Institutional Review Board of Kangbuk Samsung Hospital (KBSMC 2015-07-019). The acquisition of informed consent was waived, as we retrospectively accessed only data that were de-identified. All data were fully anonymized before our analyses.

## Results

### Baseline cross-sectional study

A total of 267,530 participants were included in analyses for evaluation of cross-sectional relationship between chronic diseases and prevalent ACD. The participants who made up of study cohort are presented in S1 Table by the year of registration. Of these, 2,071 (0.77%) had ACD at baseline. The baseline characteristics of the study participants by prevalence of prevalent ACD are presented in S2 Table. Age, HDL-C, and high sensitivity C-reactive protein were positively associated with the prevalent ACD, whereas BMI, uric acid, eGFR, fasting glucose, total cholesterol, LDL-C, triglycerides, alanine aminotransferase, aspartate aminotransferase, gamma glutamyl transaminase, and HOMA-IR were negatively associated with prevalent ACD. The proportions of male, current smoking, alcohol intake, vigorous exercise, higher education level, MeS, hypertension, chronic liver disease, and obesity were also negatively associated with prevalent ACD. The proportion of patients with CKD was positively associated with prevalent ACD.

S3 Table shows the baseline cross-sectional relationships between chronic diseases and prevalent ACD. The baseline severity of eGFR and diabetic status were significantly associated with an increased prevalence of ACD (*P*-trend < 0.001). In contrast, the baseline number of MetS traits, BP category, and low BMI category were significantly associated with a decreased prevalence of ACD (*P*-trend < 0.001). The chronic liver disease and COPD were not significantly associated with the prevalence of ACD.

In a multivariate model adjusted for age, sex, smoking, alcohol intake, physical activity, education level, center, and year of screening examination, decreased baseline eGFR and severe diabetic status were positively associated with an increased prevalence of ACD; number of MetS traits and higher levels of BP and BMI were inversely associated with prevalent ACD.

### Prospective cohort study

After excluding 2,071 subjects with baseline ACD, 265,459 subjects were included in cohort study to investigate the risk factors for incident ACD. The baseline characteristics of the cohort study participants by incident ACD are presented in Table 1. Table 2 shows the prospective associations between the chronic diseases and their severity with ACD among subjects without ACD at baseline. During 1,368,691.2 person-years of follow-up, 4,906 participants developed ACD (incidence rate 3.58 per 1,000 person-years). The average follow-up period for subjects who did not develop ACD was 5.2 years.

**Table 1:** Baseline characteristics of the study participants by incidence of anemia of chronic disease (ACD).

Characteristics	Overall	No incident ACD	Incident ACD	*P* value
Number	265,459	260,553	4,906	<0.001
Age (years)^a^	37.7 (8.0)	37.7 (8.0)	38.5 (9.0)	<0.001
Male (%)	58.3	59.0	20.1	<0.001
Current smoker (%)	24.7	25.0	8.8	<0.001
Alcohol intake (%)^c^	17.4	17.6	8.0	<0.001
Vigorous exercise (%)^d^	14.7	14.7	13.7	0.046
High education level (%)	82.5	82.6	76.7	<0.001
Chronic kidney disease (%)	0.5	0.5	2.1	<0.001
Diabetes (%)	3.1	3.0	4.8	<0.001
Metabolic syndrome (%)	13.7	13.8	8.6	<0.001
Hypertension (%)	11.5	11.5	9.3	<0.001
Chronic liver disease	30.4	30.7	15.6	<0.001
COPD (%)	1.7	1.7	1.2	0.009
Obesity (%)	28.3	28.6	14.1	<0.001
BMI (kg/m^2^)	23.3 (3.2)	23.3 (3.2)	22.0 (2.9)	<0.001
Systolic BP (mmHg)^a^	111.4 (13.5)	111.5 (13.5)	106.6 (14.4)	<0.001
Diastolic BP (mmHg)^a^	71.9 (9.9)	72.0 (9.8)	68.1 (10.1)	<0.001
Uric acid (mg/dl)^a^	5.4 (1.4)	5.4 (1.4)	4.6 (1.3)	<0.001
eGFR	93.8 (15.4)	93.8 (15.4)	94.9 (17.6)	<0.001
Hb (mg/dl)^a^	14.7 (1.4)	14.7 (1.4)	13.0 (0.9)	<0.001
Glucose (mg/dl)^a^	94.3 (14.1)	94.3 (14.0)	94.0 (20.6)	0.164
Total cholesterol (mg/dl)^a^	191.7 (33.4)	191.8 (33.4)	185.6 (33.5)	<0.001
LDL-C (mg/dl)^a^	113.5 (30.2)	113.6 (30.2)	104.8 (29.0)	<0.001
HDL-C (mg/dl)^a^	56.3 (13.8)	56.2 (13.8)	60.3 (14.1)	<0.001
Triglycerides (mg/dl)^b^	95 (67–142)	96 (67–142)	76 (57–109)	<0.001
Ferritin (mg/dl)^b^	89.0 (40.7–165.7)	90.2 (41.1–167.2)	49.9 (29.8–84.4)	<0.001
ALT (U/l)^b^	20 (14–29)	20 (14–29)	15 (12–21)	<0.001
AST (U/l)^b^	21 (18–26)	21 (18–26)	20 (16–24)	<0.001
GGT (U/l)^b^	20 (13–35)	20 (13–35)	13 (10–21)	<0.001
HOMA-IR^b^	1.59 (1.05–2.22)	1.59 (1.05–2.22)	1.51 (0.94–2.06)	<0.001
hsCRP (mg/l)^b^	0.4 (0.2–0.9)	0.4 (0.2–0.9)	0.3 (0.2–0.8)	<0.001

Data are

^a^means (standard deviation)

^b^medians (interquartile range), or percentages.

Abbreviations: ALT, alanine aminotransferase; BMI, body mass index; BP, blood pressure; HDL-C, high-density lipoprotein-cholesterol; hsCRP, high sensitivity C-reactive protein; HOMA-IR, homeostasis model assessment of insulin resistance.

^c^ ≥ 20 g of ethanol per day

^d^ ≥ 3 time per week

**Table 2:** Development of anemia of chronic disease (ACD) based on chronic diseases in the prospective cohort study.

	Person-years	Incident ACD	Incidence Density (per 1000 person-years)	Age and sex-adjusted HR (95% CI)	Multivariate adjusted HR ^a^ (95% CI)
**eGFR**					
≥60	1,362,098.0	4,801	3.5	1.00 (reference)	1.00 (reference)
30~60	6,501.0	98	15.1	3.24 (2.63–4.00)	3.93 (3.18–4.85)
<30	72.8	7	96.2	30.85 (14.66–64.93)	39.11 (18.50–82.69)
*P* for trend				<0.001	<0.001
**Diabetic status**					
Normal	908,913.4	3,102	3.4	1.00 (reference)	1.00 (reference)
Prediabetes	422,096.3	1,569	3.7	1.19 (1.12–1.26)	1.19 (1.12–1.27)
Diabetes	37,638.8	235	6.2	2.24 (1.95–2.57)	2.46 (2.14–2.84)
*P* for trend				<0.001	<0.001
**Number of Metabolic syndrome trait**					
** 0**	577,599.1	2,846	4.9	1.00 (reference)	1.00 (reference)
1	367,303.6	1,155	3.1	0.82 (0.76–0.88)	0.93 (0.87–1.00)
2	231,672.2	479	2.1	0.67 (0.60–0.74)	0.83 (0.74–0.92)
≥3	190,890.7	420	2.2	0.78 (0.70–0.87)	1.06 (0.93–1.20)
*P* for trend				<0.001	0.261
**BP category**					
Normal	814,636.7	3,648	4.5	1.00 (reference)	1.00 (reference)
Prehypertension	398,740.4	796	2.0	0.70 (0.65–0.76)	0.79 (0.73–0.86)
Hypertension	154,289.7	458	3.0	0.96 (0.86–1.07)	1.10 (0.99–0.23)
*P* for trend				<0.001	0.223
**Chronic liver disease**					
No	1,314,000.0	4,748	3.6	1.00 (reference)	1.00 (reference)
Yes	54,691.2	158	2.9	0.87 (0.74–1.02)	0.90 (0.77–1.05)
**COPD**					
No	1,335,104.6	4,776	3.6	1.00 (reference)	1.00 (reference)
Yes	21,248.0	58	2.7	0.84 (0.65–1.09)	0.87 (0.67–1.12)
**BMI category**					
<18.5	66,554.5	418	6.3	1.02 (0.92–1.13)	0.89 (0.78–1.00)
18.5~22.9	587,934.8	2,952	5.0	1.00 (reference)	1.00 (reference)
22.9~24.9	322,610.4	844	2.6	0.80 (0.74–0.86)	0.89 (0.80–0.99)
≥25	391,407.6	691	1.8	0.64 (0.59–0.70)	0.78 (0.66–0.91)
*P* for trend				<0.001	0.044

^a^ Estimated from the logistic regression model.

Multivariate model 1 was adjusted for age, sex, center, year of screening exam, smoking status, alcohol intake, physical activity and education level: Model 2: model 1 plus adjustment for obesity, chronic kidney disease, diabetes, hypertension, COPD, metabolic syndrome, and chronic liver disease Abbreviations: BMI, body mass index; CI, confidence intervals; OR, odd ratios.

**Fig 2:**
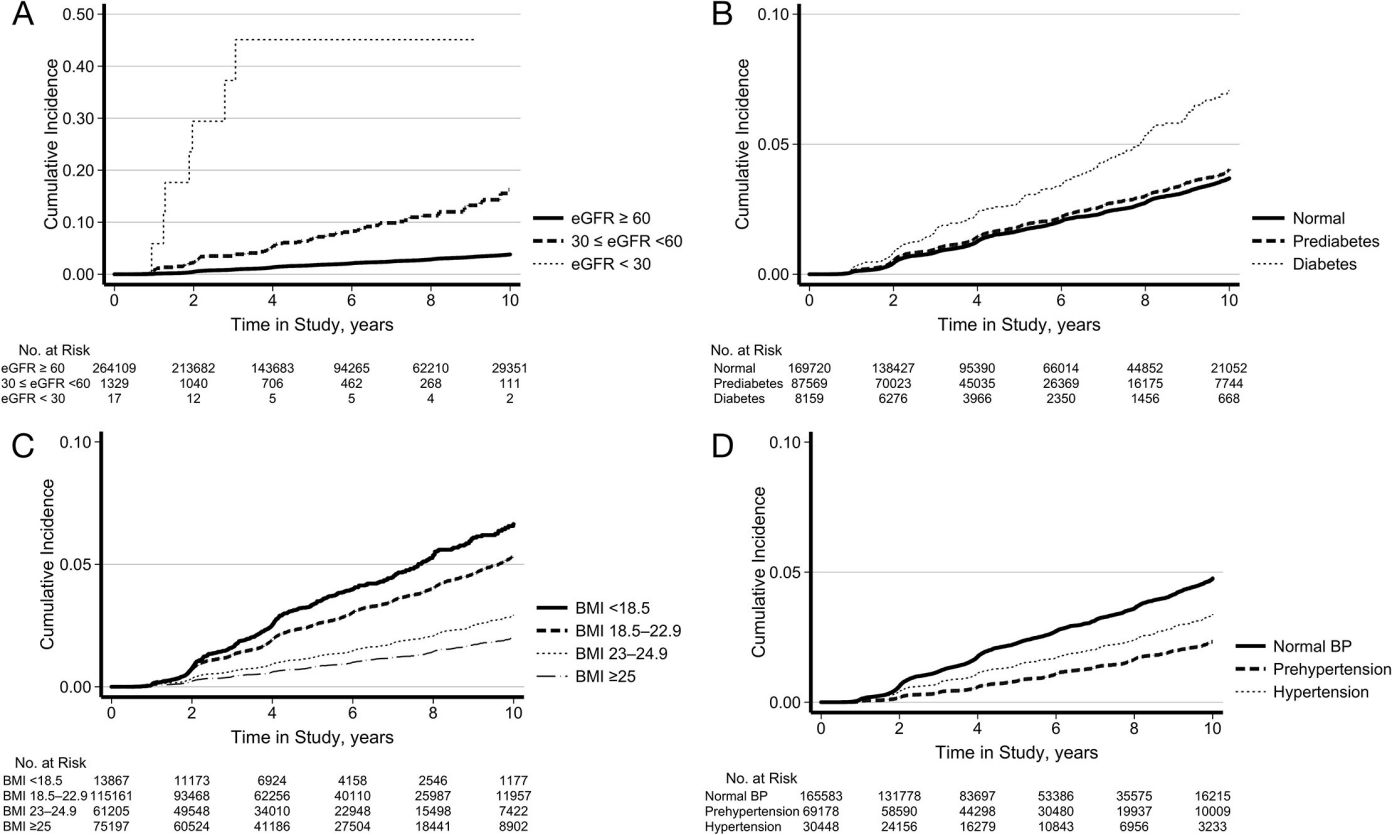
Cumulative incidence of anemia of chronic disease according to chronic kidney disease groups (A), diabetes categories (B), BMI groups (C) and BP categories (D).

Baseline severity of eGFR and diabetic status were associated with an increased risk of incident ACD in a graded and dose-response manner (*P* for trend < 0.001). Multivariable-adjusted HR (95% CI) for ACD comparing eGFR 30–60 and < 30 vs. ≥ 60 ml/min/1.73 m^2^ were 3.93 (3.18–4.85) and 39.11 (18.50–82.69), respectively (*P* for trend < 0.001). And the multivariable adjusted HR (95% CI) for ACD comparing prediabetes and diabetes vs. normal were 1.19 (1.12–1.27) and 2.46 (2.14–2.84), respectively (*P* for trend < 0.001). Increasing BMI was inversely associated with incident ACD. HR (95% CI) for ACD comparing BMIs of <18.5, 23–24.9, and >25 vs. 18.5–22.9 kg/m^2^ were 0.89 (0.78–1.00), 0.89 (0.80–0.99) and 0.78 (0.66–0.91), respectively. Prehypertension was associated with a decreased risk of ACD with corresponding HR (95% CI) of 0.79 (0.73–0.86). The cumulative incidence of ACD was displayed in Fig 2 by CKD groups (Fig 2A), diabetes categories (Fig 2B), BMI groups (Fig 2C) and BP categories (Fig 2D). Metabolic syndrome, hypertension, chronic liver disease, and COPD were not associated with the incidence of ACD in multivariate analyses.

## Discussion

In this large cohort study of young and middle-aged Korean men and women, both the cross-sectional and cohort analyses demonstrated that decreased eGFR and severe diabetic status were associated with increased risk of ACD. Conversely, higher BMI categories were associated with a decreased risk of developing ACD in dose-response manner. These associations persisted even after adjusting for possible confounders. Our results provide information about the relative risk for developing ACD among patients with various common chronic diseases.

In our study of 267,530 participants, the proportion of those with ACD was 0.77% in overall population, 0.9% of participants with diabetes and 6.7% of participants with eGFR < 60 ml/min/1.73 m^2^. However, previous studies of patients with diabetes or CKD reported a higher prevalence of anemia of 10–20%.[10–13] Our lower prevalence of ACD is partly explained by the following reasons: previous studies included nutritional anemia in the anemia outcome and reported a higher prevalence, and chronic diseases in hospital populations from other studies were more severe than in the general population of our study.

Previous studies have shown an association between eGFR, diabetes status and ACD.[10–13] Diabetes and CKD are considered chronic inflammatory states characterized by an increased level of pro-inflammatory cytokines involved in erythroid progenitor cells.[24, 25] In our study, subjects with eGFR < 30 ml/min/1.73 m^2^ had almost 40 fold increased risk of developing ACD compared with those with a eGFR ≥ 60 ml/min/1.73 m^2^. The risk of ACD in prediabetic and diabetic participants increased by 1.19 and 2.46 times compared to euglycemic participants, respectively. In CKD or diabetes, incident ACD increased as severity of these diseases increased. Contrary to our expectation, our study found that obese subjects and/or those with prehypertension had a lower risk of ACD. Given that obesity is characterized by chronic low-grade inflammation,[26, 27] a previous study also hypothesized that hemoglobin concentration might be lower in individuals with overweight or obesity.[28] However, this cross-sectional study using data from the third National Health and Nutrition Examination Survey (NHANES III) in a US population reported that overweight (BMI 25–29.9 kg/m^2^) and obese (BMI > 30 kg/m^2^) subjects were not associated with anemia compared with normal-weight subjects, while increasing BMI was associated with reduced transferrin saturation and higher serum ferritin, suggesting mechanisms of obesity-related inflammation. A recent systematic review summarized that obese subjects tend to have higher hemoglobin levels than non-obese subjects,[29] which is consistent with our findings.

The mechanisms underlying the inverse association between obesity and the risk of developing ACD are unknown. Therefore, we hypothesized the following reasons. First, subjects who might have developed obesity-induced iron deficiency anemia (IDA) were not included in our study population. Because obesity-associated inflammation affects iron homeostasis and results in an iron deficiency[30] and our study population excluded subjects with IDA. In turn, obese subjects were less likely to experience anemic outcomes. Second, obese subjects have more comorbidities, which may increase hemoglobin. Obese subjects are more likely to have obstructive sleep apnea and other obesity-related respiratory disorders, which result in chronic tissue hypoxia and lead to polycythemia.[31, 32] Third, obese subjects are less likely to be malnourished, because excessive caloric intake can develop into obesity.[26] Therefore, adequate or overnutrition in obese subjects might be associated with a reduced risk of anemia.

Several limitations of this study should be discussed. First, although we identified ACD cases after excluding nutritional anemia or possible blood loss, we may have included unexplained anemia without a proven etiology or clonal anemia.[33] However, given the younger age of our study population, the proportion of unexplained or clonal anemia cases would be minimal. Second, we have no data regarding hepcidin, a key ACD regulatory hormone and proinflammatory factors. Further mechanistic studies are required to elucidate the association between chronic diseases and ACD. Third, our findings are limited by selection bias of case definition. For example, the definition of chronic liver disease could be incomplete without consideration of laboratory findings (liver enzymes, albumin, prothrombin time). The etiology of macrocytic anemia includes chronic illness other than nutritional anemia. [20] However, the number of macrocytic anemia was very low (0.04%) and the impact of selection bias would be minimal. Finally, our study data included young and middle-aged Korean subjects who regularly attended health screening examinations, which could limit the generalizability of our results to other populations with different characteristics of age and race/ethnicity. However, our study provides reliable estimates of ACD risk because of the large sample size, the use of well-defined measurements, and the prospective nature of the cohort study.

This is the one of the largest cohort studies demonstrating a prospective association between chronic diseases and the incidence of ACD. Our cross-sectional and cohort studies identified that prehypertension and increasing BMI are independently associated with a decreased risk of ACD. Although the relationship between CKD or diabetes and anemia is well known, the negative relationships of obesity and prehypertension for incident anemia are novel and interesting findings. Anemia is a non-negligible, but unrecognizable risk. These data will allow clinicians to identify at-risk subjects for intervention. Further studies are warranted to confirm our results.

## Supporting information

S1 Table The participants who made up the KSHS cohort by the year of registration. (DOCX)

S2 Table Baseline characteristics of study participants by prevalence of anemia of chronic disease (ACD). (DOCX)

S3 Table Odds ratios (95% CI) by chronic disease and the prevalence of anemia of chronic disease (ACD) in baseline cross-sectional study. (DOCX)
