# Supplementary material for: Risk factors for incident anemia of chronic diseases: A cohort study
Source: PLoS One. 2019 May 6;14(5):e0216062. doi: 10.1371/journal.pone.0216062 (PMC6502324; doi:10.1371/journal.pone.0216062)
Supplement: S1 Table — (DOCX) [file pone.0216062.s001.docx]

**Supporting information**

**S1 Table. The participants who made up the KSHS cohort by the year of registration**

| Year of registration | Number (%) | Cumulative proportion |
| --- | --- | --- |
| 2005 | 46,619 (17.4) | 17.4% |
| 2006 | 35,989 (13.5) | 30.9% |
| 2007 | 20,129 (7.5%) | 38.4% |
| 2008 | 17,304 (6.5%) | 44.9% |
| 2009 | 17,056 (6.4%) | 51.3% |
| 2010 | 25,464 (9.5%) | 60.8% |
| 2011 | 25,998 (9.7%) | 70.5% |
| 2012 | 24,879 (9.3%) | 79.8% |
| 2013 | 26,695 (10.0%) | 89.8% |
| 2014 | 20,508 (7.7%) | 97.4% |
| 2015 | 6,889 (2.6%) | 100.0% |
|  |  |  |
