# Supplementary material for: Risk factors for incident anemia of chronic diseases: A cohort study
Source: PLoS One. 2019 May 6;14(5):e0216062. doi: 10.1371/journal.pone.0216062 (PMC6502324; doi:10.1371/journal.pone.0216062)
Supplement: S2 Table — (DOCX) [file pone.0216062.s002.docx]

**S2 Table. Baseline characteristics of study participants by prevalence of anemia of chronic disease (ACD)**

| Characteristics | Overall | No prevalent ACD | prevalent ACD | *P* value |
| --- | --- | --- | --- | --- |
| Number | 267,530 | 265,459 | 2,071 | <0.001 |
| Age (years)^a^ | 37.7 (8.0) | 37.7 (8.0) | 38.9 (10.0) | <0.001 |
| Male (%) | 57.9 | 58.3 | 13.1 | <0.001 |
| Current smoker (%) | 24.5 | 24.7 | 4.8 | <0.001 |
| Alcohol intake (%)^b^ | 17.4 | 17.4 | 5.6 | <0.001 |
| Vigorous exercise (%)^c^ | 14.7 | 14.7 | 12.8 | 0.014 |
| High education level (%)^d^ | 82.4 | 82.5 | 78.9 | <0.001 |
| Chronic kidney disease (%) | 0.5 | 0.5 | 4.7 | <0.001 |
| Diabetes (%) | 3.1 | 3.1 | 3.7 | 0.083 |
| Metabolic syndrome (%) | 13.7 | 13.7 | 4.8 | <0.001 |
| Hypertension (%) | 11.5 | 11.5 | 8.1 | <0.001 |
| Chronic liver disease | 30.3 | 30.4 | 11.4 | <0.001 |
| COPD (%) | 1.7 | 1.7 | 1.5 | 0.577 |
| Obesity (%) | 28.2 | 28.3 | 10.0 | <0.001 |
| BMI (kg/m^2^) | 23.3 (3.2) | 23.3 (3.2) | 21.6 (2.7) | <0.001 |
| Systolic BP (mmHg)^a^ | 111.4 (13.6) | 11.4 (13.5) | 102.9 (13.7) | <0.001 |
| Diastolic BP (mmHg)^a^ | 71.9 (9.9) | 71.9 (9.9) | 64.6 (9.5) | <0.001 |
| Uric acid (mg/dl)^a^ | 5.4 (1.4) | 5.4 (1.4) | 4.3 (1.4) | <0.001 |
| eGFR | 93.8 (15.5) | 93.8 (15.4) | 99.7 (22.8) | <0.001 |
| Hb (mg/dl)^a^ | 14.7 (1.4) | 14.7 (1.4) | 11.7 (0.5) | <0.001 |
| Glucose (mg/dl)^a^ | 94.3 (14.1) | 94.3 (14.1) | 90.3 (12.8) | <0.001 |
| Total cholesterol (mg/dl)^a^ | 191.6 (33.5) | 191.7 (33.4) | 176.8 (33.3) | <0.001 |
| LDL-C (mg/dl)^a^ | 113.4 (30.2) | 113.5 (30.2) | 99.4 (27.9) | <0.001 |
| HDL-C (mg/dl)^a^ | 56.3 (13.8) | 56.3 (13.8) | 60.6 (15.2) | <0.001 |
| Triglycerides (mg/dl)^e^ | 95 (67-142) | 95 (67-142) | 70 (52-101) | <0.001 |
| Ferritin (mg/dl)^e^ | 88.5 (40.7-165.2) | 89.0 (40.7-165.7) | 55.8 (40.4-89.5) | <0.001 |
| ALT (U/l)^e^ | 20 (14-29) | 20 (14-29) | 14 (11-19) | <0.001 |
| AST (U/l)^e^ | 21 (17-26) | 21 (18-26) | 18 (15-22) | <0.001 |
| GGT (U/l)^e^ | 20 (13-35) | 20 (13-35) | 12 (9-17) | <0.001 |
| HOMA-IR^e^ | 1.58 (1.04-2.21) | 1.59 (1.05-2.22) | 1.28 (0.68-1.69) | <0.001 |
| hsCRP (mg/l)^e^ | 0.4 (0.2-0.9) | 0.4 (0.2-0.9) | 0.4 (0.2-1.1) | 0.001 |
| Data are ^a^means (standard deviation), ^b^≥ 20 g of ethanol per day, ^c^≥ 3 times per week, ^d^ ≥ College graduate, ^e^medians (interquartile range), or percentages.  Abbreviations: ALT, alanine aminotransferase; BMI, body mass index; BP, blood pressure; HDL-C, high-density lipoprotein-cholesterol; hsCRP, high sensitivity C-reactive protein; HOMA-IR, homeostasis model assessment of insulin resistance. | | | | |
