# Supplementary material for: Risk factors for incident anemia of chronic diseases: A cohort study
Source: PLoS One. 2019 May 6;14(5):e0216062. doi: 10.1371/journal.pone.0216062 (PMC6502324; doi:10.1371/journal.pone.0216062)
Supplement: S3 Table — (DOCX) [file pone.0216062.s003.docx]

**S3 Table. Odds ratios^a^ (95% CI) by chronic disease and the prevalence of anemia of chronic disease (ACD) in baseline cross-sectional study**

|  | Person | Prevalent ACD | Prevalence rate, % | Age and sex-adjusted OR  (95% CI) | Multivariate adjusted OR^a^  (95% CI) |
| --- | --- | --- | --- | --- | --- |
| **eGFR** |  |  |  |  |  |
| ≥60 | 266,083 | 1,974 | 0.7 | 1.00 (reference) | 1.00 (reference) |
| 30~60 | 1,390 | 61 | 4.4 | 5.44 (4.10-7.22) | 6.67 (5.01-8.88) |
| <30 | 53 | 36 | 67.9 | 621.08 (325.26-1185.92) | 585.93 (305.10-1125.27) |
| *P* for trend |  |  |  | <0.001 | <0.001 |
| **Diabetic status** |  |  |  |  |  |
| Normal | 170,992 | 1.272 | 0.7 | 1.00 (reference) | 1.00 (reference) |
| Prediabetes | 88,292 | 723 | 0.8 | 1.22 (1.11-1.34) | 1.16 (1.06-1.28) |
| Diabetes | 8,235 | 76 | 0.9 | 1.61 (1.26-2.06) | 1.62 (1.27-2.07) |
| *P* for trend |  |  |  | <0.001 | <0.001 |
| **Number of Metabolic syndrome trait** |  |  |  |  |  |
| 0 | 117,240 | 1,212 | 1.0 | 1.00 (reference) | 1.00 (reference) |
| 1 | 69,854 | 563 | 0.8 | 1.05 (0.94-1.16) | 1.12 (1.01-1.24) |
| 2 | 43,610 | 194 | 0.4 | 0.73 (0.62-0.85) | 0.80 (0.68-0.93) |
| ≥3 | 36,537 | 99 | 0.3 | 0.50 (0.40-0.62) | 0.56 (0.45-0.69) |
| *P* for trend |  |  |  | <0.001 | <0.001 |
| **BP category** |  |  |  |  |  |
| Normal | 167,271 | 1,688 | 1.0 | 1.00 (reference) | 1.00 (reference) |
| Prehypertension | 69,390 | 212 | 0.3 | 0.50 (0.43-0.58) | 0.57 (0.49-0.66) |
| Hypertension | 30,616 | 168 | 0.6 | 0.77 (0.65-0.92) | 0.84 (0.70-1.01) |
| *P* for trend |  |  |  | <0.001 | <0.001 |
| **Chronic liver disease** |  |  |  |  |  |
| No | 257,288 | 2,011 | 0.8 | 1.00 (reference) | 1.00 (reference) |
| Yes | 10,242 | 60 | 0.6 | 0.83 (0.64-1.07) | 0.84 (0.65-1.08) |
| **COPD** |  |  |  |  |  |
| No | 260,807 | 2,011 | 0.8 | 1.00 (reference) | 1.00 (reference) |
| Yes | 4,446 | 31 | 0.7 | 1.12 (0.78-1.61) | 1.16 (0.81-1.66) |
| **BMI category** |  |  |  |  |  |
| <18.5 | 14,072 | 205 | 1.5 | 1.02 (0.88-1.19) | 0.97 (0.84-1.13) |
| 18.5~22.9 | 116,492 | 1,331 | 1.1 | 1.00 (reference) | 1.00 (reference) |
| 22.9~24.9 | 61,532 | 327 | 0.5 | 0.75 (0.66-0.85) | 0.78 (0.69-0.88) |
| ≥25 | 75,405 | 208 | 0.3 | 0.48 (0.41-0.56) | 0.50 (0.43-0.58) |
| *P* for trend |  |  |  | <0.001 | <0.001 |
| ^a^ Estimated from logistic regression model.  Multivariate model 1 was adjusted for age, sex, center, year of screening exam, smoking status, alcohol intake, physical activity and education level: Model 2: model 1 plus adjustment for obesity, chronic kidney disease, diabetes, hypertension, COPD, metabolic syndrome, and chronic liver disease Abbreviations: BMI, body mass index; CI, confidence intervals; OR, odd ratios. | | | | | |
